# Environmental exposure to arsenic and chromium in an industrial area

**DOI:** 10.1007/s11356-017-8827-6

**Published:** 2017-03-20

**Authors:** Luigi Vimercati, Maria F Gatti, Tommaso Gagliardi, Francesco Cuccaro, Luigi De Maria, Antonio Caputi, Marco Quarato, Antonio Baldassarre

**Affiliations:** 1 Interdisciplinary Department of Medicine, Occupational Medicine “B. Ramazzini”, University of Bari Medical School, Giulio Cesare Square 11, 70124 Bari, Italy; 2 Health Local Unit of Barletta-Andria-Trani, 76121 Barletta, Italy

**Keywords:** Arsenic, Chromium, Carcinogens, Environmental exposure, Biological monitoring, Public health

## Abstract

Arsenic and chromium are widespread environmental contaminants that affect global health due to their toxicity and carcinogenicity. To date, few studies have investigated exposure to arsenic and chromium in a population residing in a high-risk environmental area. The aim of this study is to evaluate the exposure to arsenic and chromium in the general population with no occupational exposure to these metals, resident in the industrial area of Taranto, Southern Italy, through biological monitoring techniques. We measured the levels of chromium, inorganic arsenic, and methylated metabolites, in the urine samples of 279 subjects residing in Taranto and neighboring areas. Qualified health staff administered a standardized structured questionnaire investigating lifestyle habits and controlling for confounding factors. The biological monitoring data showed high urinary concentrations of both the heavy metals investigated, particularly Cr. On this basis, it will be necessary to carry out an organized environmental monitoring program, taking into consideration all exposure routes so as to correlate the environmental concentrations of these metals with the biomonitoring results.

## Introduction

Arsenic and chromium (Cr) are widespread environmental contaminants that affect global health due to their toxicity and carcinogenicity (Centeno et al. 2006; IARC 2012a, 2012b; Tsai et al. 2003; Lai et al. 1994; Lee et al. 2002; Chakraborti et al. 2003). Both exist ubiquitously in the environment and they are commonly present in air, water, soil, and sediments and could find their routes into the human body through inhalation, ingestion, and skin absorption (ATSDR, Agency for Toxic Substances and Disease Registry 2007).

Arsenic exists in organic and inorganic forms and in different oxidation or valence states. The valence states of arsenic compounds relevant to human health are the trivalent (AsIII) and pentavalent (AsV) states. These arsenic species include arsenates (compounds containing AsO_4_^3−^), arsenites (compounds containing AsO_3_^3−^), and the mono-methyl (MMA) and di-methyl (DMA) metabolites. Arsenic species in the trivalent (III) state including arsenous acid (commonly arsenite), dimethylarsinous acid (DMAIII), and monomethylarsonous acid (MMAIII) are estimated more toxic at lower doses than those of other arsenic species (ATSDR 2007; Drobna et al. 2009). Results from animal and epidemiological studies have shown that inorganic arsenic (iAs) compounds can be categorized as carcinogens (group 1) or potential carcinogens (group 2B) such as DMA and MMA, while arsenobetaine and other organoarsenicals have not been categorized as carcinogens (group 3) (IARC 2012a, 2012b).

The primary source of arsenic exposure in human is drinking water (NRC 1999; NRC 2001; Smith et al. 2002; Watanabe et al. 2003). Organic arsenic compounds (e.g., arsenobetaine) are mainly found in fish, which thus may give rise to human exposure, whereas inorganic arsenic is found in groundwater used for drinking in different areas of the world (such as Asia and South America), although it has been long recognized that arsenic exposure from drinking water is causally related to cancer in the lungs, kidney, bladder, and skin (WHO 2001). Water reservoirs can be contaminated by oil refining wastes: indeed, conventional waste-treatment techniques are not effective in the removal of the same arsenical species contained in crude oil (e.g., As(III) and As(V)) (Tonietto et al. 2010). In addition, contaminated soils are a source of arsenic exposure (WHO 2001). Soil can be contaminated by arsenic-rich steel plants’ emissions, due to the melting of metal scrap containing this metalloid in blast furnaces to obtain recycled iron. Neighboring land may be polluted for decades after plant closure (Lambert et al. 2011).

Cr(0), Cr(III), and Cr(VI) are used commercially and are present in the environment. Cr(0) is mainly in its metallic form as a component of iron-based alloys such as stainless steel.

Trivalent chromium (Cr(III)) is primarily of geological origin and hexavalent chromium (Cr(VI)) is derived mainly from industrial processes (Zhitkovich 2011), but can also originate from the oxidation of naturally occurring Cr(III) by minerals containing Mn-oxides (Oze et al. 2007).

Inter alia, since 1960s, Cr(0) is mainly used in its metallic form by steel factories as a component of iron-based alloys such as stainless steel and tin-free steel, less expensive than tin steel. During these processes, metallic powder containing chromium ions and chromium oxides is produced and, despite the presence of particular filters, it is poured into the air. Moreover, factories’ blast furnaces melt metal scrap containing chromium and other ions to obtain steel. Due to high temperature, Cr(III) and Cr(VI) evaporate and are output via the furnaces’ chimneys (Nicodemi 1994). Chromium also is a waste product in oil refining processes (on average, 27–80 mg per kg of oily sludge): a wrong waste management could lead to a substantial soil pollution around the refinery plant (Bhattacharyya and Shekdar 2003). Finally, cement factories produce dusts during raw material processing, grinding of clinker, and packaging of finished product: this matter is composed of several elements, among which different species of chromium and arsenic and it is deposited to many hundreds of meters from the site of release (Gbadebo and Bankole 2007).

In food and dietary supplements, chromium is mainly Cr(III) and is considered to play a key role in human metabolism, otherwise in drinking, water is primarily Cr(VI).

In contrast with arsenic, is not so clear if chromium can cause cancer following ingestion via drinking water. According to this hypothesis, two ecological studies conducted in China (IARC 1990; Beaumont et al. 2008) and in Greece (Linos et al. 2011) estimated lung and stomach cancer mortality associated with prolonged oral consumption of water contaminated with Cr(VI).

Like for arsenic, it has long been established that inhalation of chromium, in particular hexavalent chromium (CrVI), can cause human lung cancer (IARC 1990).

The specific mechanism of chromium carcinogenicity remains unclear; however, there is an abundance of data supporting the genotoxicity and mutagenicity of Cr(VI) in vivo and in vitro. Particularly, chromium in its hexavalent form is considered to be a pro-carcinogen. Cr(VI) manages to enter the cell through molecular mimicry mechanism as an oxyanion followed by its metabolic reduction to Cr(V), Cr(IV), and to the final reduced trivalent form. These reduced forms have been shown to induce a wide range of genomic DNA damage, which may lead to DNA replication inhibition (Salnikow and Zhitkovich 2008; Alexander and Aaseth 1995; O’Brien et al. 2001). Moreover, Cr(VI) is thought to be able to induce DNA double-stranded breaks selectively on euchromatin and to accumulate ubiquitinated forms of histone H2AX: these two kinds of damage lead to suppressed upregulation of inducible genes and help to explain the high genotoxic potential of this metal (DeLoughery et al. 2015).

As for chromium, the precise mechanisms that acute or chronic exposure to arsenic performs to induce cancer are not yet understood. Recent studies have shown that the toxicity of arsenic depends on several factors: exposure amount, length, and frequency, biological species, age, sex, individual susceptibility, genetics, and nutrition (Abernathy et al. 1999).

Different hypotheses regarding the toxic mechanism behind arsenic have been suggested, including induced chromosome abnormalities, promotion/progression, oxidative stress, suppression of p53, altered DNA repair, enhanced cell proliferation, altered DNA methylation patterns, altered growth factors, and gene amplification (Hong et al. 2014).

The aim of this study was to assess arsenic and chromium exposure in the general population with no occupational exposure to these metals, resident in the municipalities of Taranto and Statte, site of a large integrated cycle steel foundry, a refinery, and a cement factory, and in the municipality of Laterza, 54-km driving distance from Taranto, considered as a non-polluted area because no significant industrial plants are present.

In fact, the land close to Taranto industrial plant is polluted by many heavy metals, as well as PAHs (Campo et al. 2012): the water samples also taken from aquifers contain high arsenic and chromium concentrations (ARPA 2009). The steel plant in Taranto, for 2005, has emitted into the air 3800.8 kg of chromium and compounds, into the water 20,407.3 kg of chromium and compounds, and 1172.1 kg of arsenic and compounds (APAT - INES 2005). Moreover, in Taranto Gulf, soil contamination is partially linked to air pollution and both could be not homogeneously distributed in closer sites (e.g., Paolo VI, Statte) due to meteorological conditions (Mangia et al. 2013).

## Materials and methods

Between January 2010 and April 2012, a cross-sectional study was conducted to measure the urinary excretion of inorganic arsenic and its methylated metabolites monomethyl arsenic acid (MMA) and dimethylarsinic acid (DMA), chromium as well as urinary creatinine, which was used both to confirm the acceptability of urine samples and to adjust the metal concentrations.

The analysis of the urine samples was performed by atomic absorption spectrophotometry (PerkinElmer Corp.—Model 5100 PC—PerkinElmer Inc.—Wellesley, MA, USA), employing the hydrides (arsine) generation technique for determining As and the graphite furnace method for Cr according to the NIOSH analytical methods (NIOSH 2003); an automated kinetic Jaffe technique using alkaline picric acid was used to measure creatinine. Urine samples were not processed for metal concentrations if the creatinine excretion was not within the range of 0.3–3.0 g/L (ACGIH 2010).

An internal quality control (IQC), according to manufacturer instructions, was used systematically to verify the reproducibility and the repeatability of the data that were obtained using the standard curve prepared by the operator.

The research was conducted with 350 subjects residing in Taranto and the surrounding area for at least 10 years; they were randomly selected from the Regional Assisted Care Registry to reduce the possibility of bias in self-selection. The response rate was high (93.1%).

All of the subjects were contacted in accordance with procedures agreed upon by local general practitioners, who had previously been invited to a dedicated meeting at which they were fully informed about the aims of the study and asked whether they would be willing to collaborate. All subjects agreed to the processing of their personal data and understood that this information was categorized as “sensitive data”. All subjects were informed that data from the research protocol would be treated in an anonymous and collective way, with scientific methods and for scientific purposes in accordance with the principles of the Helsinki Declaration.

After obtaining informed consent from each participant qualified health staff it was administered a standardized structured questionnaire. Each questionnaire included personal data of the study participants and information of lifestyle habits. In particular, the questionnaire has investigated the residential history, housing exposure (intensity of car traffic, the presence of fireplace inside their home, proximity to industrial areas), environmental exposure (use of pesticides, paints, wood preservatives), and occupational exposure (company, type of job, use of PPE). In addition, they were investigated regarding eating habits especially if they had consumed seafood in the last 48–72 h before urine collection and were evaluated with other confounding factors including quantity and type of water consumed (tap or bottled mineral water) and smoking habits (type, cigarettes/day, years of smoking, use of cigars, pipe). The questionnaire also investigates the presence of diseases and use of drugs.

First-void urine (FVU) was sampled from each participant into clean conical 50-mL polypropylene tubes, which were then immediately sealed with O-ring screw caps and packed into coolers with frozen ice packs. Samples were sent to the laboratory and then stored at −20 °C and analyzed within 1 month.

After analysis, we excluded 47 (13.4%) subjects from the study because of creatinine excretion values <0.3 g/L or >3.0 g/L.

After these exclusions, the final sample consisted of 279 subjects, including 135 males and 144 females aged between 18 and 77 years (mean age 46.0 ± 13.12 SD). Of the 279 study subjects, 179 resided in the city of Taranto; they were subdivided into three district areas: “Paolo VI” (*N* 39), “Tamburi—Old Town” (*N* 50), or “New Town” (*N* 90). A total of 55 subjects were residents of the nearby Statte municipality and 45 resided in the Laterza municipality (Table 1).

**Table 1:** Flying distance from the industrial site

Laterza	34 km
Statte	2.3 km
Paolo VI	3 km
Tamburi—Old Town	200 m
New Town	2.7 km

Comparisons among groups were made employing non-parametric techniques (rank sum Wilcoxon-Mann-Whitney test and Kruskal-Wallis test). We also performed a multivariate analysis through a linear regression model, investigating the association of the urinary concentrations of As and Cr with the explanatory variables obtained by questionnaire. The main variables included in the model were age, sex, body mass index, drinking water, smoking habits, city of residence, dwelling site, consumption of fish, crustaceans, and shellfish in the 48–72 h before collection, presence of dental fillings, and use of fireplaces in homes.

A *p* value ≤0.05 was considered significant. Statistical analysis was conducted using packages SAS (v. 9.0) and STATA (v. 11).

## Results

Table 2 shows the urinary levels of iAs + MMA + DMA, and Cr measured in the overall population of 279 subjects residing in Taranto and neighboring areas.

**Table 2:** Mean, median, and 95th percentile of the urinary levels of As and Cr (μg/L) by municipality

Municipality	Metal	mean	SD	p5	p50 (median)	p95	*n*	SIVR reference values
Whole study population	iAs + MMA + DMA	6.1	8.6	1.4	3.8	16.8*	279	2.0–15
	Cr	0.5	0.5	0.1	0.3	1.3*	279	0.05–0.35
Taranto	iAs + MMA + DMA	5.2	8	1.5	3.8	11.1	179	2.0–15
	Cr	0.4	0.3	0.1	0.3	1*	179	0.05–0.35
Laterza	iAs + MMA + DMA	3.2	2.3	0.9	2.7	8.5	45	2.0–15
	Cr	0.4	0.4	0.1	0.3	1.2*	45	0.05–0.35
Statte	iAs + MMA + DMA	11.5	11	2.5	8.8	27.1*	55	2.0–15
	Cr	0.9	1	0.2	0.5	2.5*	55	0.05–0.35

*Statistically significant

It was carried out a comparison of the urinary Cr and As in study groups with the range proposed by the Italian Reference Values Society (SIVR).

Table 3 shows the urinary levels of iAs + MMA + DMA, and Cr measured in the different districts of Taranto (Paolo VI, Tamburi-Old Town, New Town).

**Table 3:** Mean, median, and 95th percentile of the urinary levels of As and Cr (μg/L) in the different districts in the city of Taranto

District	Metal	mean	SD	p5	p50 (median)	p95	n	SIVR reference values
New Town	iAs + MMA + DMA	4.3	2.4	1.7	3.8	9.7	90	2.0–15
	Cr	0.3	0.3	0.1	0.3	0.9*	90	0.05–0.35
Paolo IV	iAs + MMA + DMA	4	3.7	1.4	2.7	9.1	39	2.0–15
	Cr	0.4	0.2	0.1	0.4	0.8*	39	0.05–0.35
Tamburi—Old Town	iAs + MMA + DMA	7.8	14.3	1.4	4.6	14.3	50	2.0–15
	Cr	0.4	0.4	0.1	0.3	1.2*	50	0.05–0.35

*Statistically significant

### Urinary chromium

The median value was 0.3 μg/L, which was comparable to the upper limit of the range proposed by the SIVR. The 95th percentile was significantly above the reference value limits (Table 2).

The differences in the measured median values among the municipalities were significant, with the highest values found in Statte (Table 2). In the different districts of Taranto, the highest median values of urinary concentrations were found in the Paolo VI district (Table 3).

The median values of urinary chromium were comparable in both sexes, in all age classes, among smokers and no smokers, regardless of whether they drank tap or bottled mineral water, and seafood consumption (Table 4).

**Table 4:** Urinary excretion of As and Cr metals (μg/L) in relation to the variables listed

	Sex		Smoking habit		Water		Seafood consumption	
	Male	Female	Yes	No	Tap	Bottled	Yes	No
IAs + MMA + DMA	4.1 (135)	3.8 (144)	4.1 (73)	3.8 (206)	3.6* (93)	2.5 (186)	9.8* (35)	3.8 (244)
Cr	0.4 (135)	0.3 (144)	0.5 (73)	0.3 (206)	0.5 (93)	0.4 (186)	0.4 (35)	0.3 (244)

*Statistically significant

() = *n* value

In our study, we investigated the association between having a fireplace in home and the urinary excretion of Cr, without finding any association.

The multivariate analysis showed the following results. For Cr, we found an association with the city of residence; specifically, we found a higher concentration in the people living in Statte vs Taranto (*p* = 0.001), and this analysis is not influenced by principal investigated confounding factors (Table 5).

**Table 5:** Urinary excretion of As and Cr metals (μg/L) in relation to the variables listed in Statte municipality

	Sex		Smoking habit		Water		Seafood consumption	
	Male	Female	Yes	No	Tap	Bottled	Yes	No
IAs + MMA + DMA	10.4 (27)	7.0 (28)	8.2 (16)	9.9 (39)	8.4 (28)	9.4 (27)	11.8 (4)	8.5 (51)
Cr	0.6 (27)	0.5 (28)	0.5 (16)	0.5 (39)	0.5 (28)	0.7 (27)	1 (4)	0.5 (51)

() = *n* value

### Urinary inorganic arsenic and methylated metabolites

The median urinary concentration in the entire study population was within the SIVR reference limit whereas the 95th percentile was higher than that of the upper limit (Table 2). Considering the municipalities, the 95th percentile was higher than the reference value only in Statte (Table 2). Moreover, the median urinary concentration of iAs + MMA + DMA was significantly higher in Statte than that in Taranto or Laterza, although it was still within the range limits (Table 2). In the different districts in the city of Taranto, the 95th percentile and median urinary values remained within range limits (Table 3).

When analyzing the urinary excretion of iAs + MMA + DMA in relation to the variables considered in the study population, similar median values were obtained in both sexes. In subjects who drank tap water, urinary iAs + MMA + DMA values (3.6 μg/L) were higher than in those who drank bottled mineral water (2.5 μg/L). Slightly higher values were found in smokers (4.1 μg/L) than those in non-smokers (3.8 μg/L). Statistically significant differences were found when comparing the urinary concentrations in those who had eaten shellfish and/or seafood in the 48–72 h before sampling (9.8 vs 3.8 μg/L) (Table 4).

In our study, no association between the use of pesticides and urinary concentrations of As was found. Moreover, we investigated the association between having a fireplace in home and the urinary excretion of As, without finding any association.

The multivariate analysis showed the following results. For As, we found an association with the city of residence; specifically, we found a higher concentration in the people living in Statte vs Taranto (*p* = 0.001) and this analysis is not influenced by principal investigated confounding factors (Table 5). We also found a lower concentration in the people living in Laterza vs Taranto (*p* = 0.037) and a statistically significant association with the consumption of crustaceans in the 48–72 h before collection (*p* = 0.019).

## Discussion

To date, few studies have investigated exposure to arsenic and chromium in a population residing in a high-risk environmental area such as Taranto, Apulia Region (Southern Italy) (Iavarone et al. 2012). Many years have passed since the WHO first included the Taranto area among those at high environmental risk and underlined the increased mortality rates, as compared to Italy as a whole, for bladder, liver, and lung cancer, as well as cancer of the pleura and non-Hodgkin lymphoma (WHO 1997).

The median value for chromium (0.3 μg/L) was the upper limit value of the relative SIVR range, while the 95th percentile was actually higher than the proposed SIVR upper limit. There were no significant differences in urinary excretion by sex, age, type of water drunk, and number of smoked cigarettes, unlike in other reports in literature (EPA 1984; SIVR 2011; Zhitkovich 2002). Moreover, our study found higher urinary levels of chromium and arsenic in people living close to industrial plants.

It has been generally accepted that low or moderate doses of orally ingested Cr(VI) are non-carcinogenic, but, more recently, the potential risks of Cr(VI) exposure by ingestion in drinking water have come under increased scrutiny (Nickens et al. 2010).

The chromium concentration limit in drinking water applied both in Italy (Legislative Decree no. 31/2001) and in the USA. (US Environmental Protection Agency—EPA) is 50 μg/L (ATSDR 2007). However, in a study conducted in California (USA), 38% of municipal sources of drinking water reportedly showed higher levels of chromium (VI) than the detection limit of 1 μg/L (Sedman et al. 2006).

Tobacco smoke is known to contain chromium (VI), and indoor air polluted by cigarette smoke can contain hundreds of times the amount of chromium (VI) found in outdoor air (IARC 2012a, 2012b). However, other different exposure routes, such as transdermal way (as well as inhalation and ingestion), are possible but their contribution to overall chromium intake is not clear and cannot be investigated in this study.

The subjects who had eaten seafood and/or shellfish 48–72 h before urine collection had higher levels of urinary excretion of arsenic (9.8 vs 3.8 μg/L). In fact, diet is the main source of non-occupational exposure to arsenic (Vimercati et al. 2009). Foods with the highest content of arsenic include some marine organisms, such as shellfish and crustaceans (Argese et al. 2005; Fattorini et al. 2004; Lopez et al. 1994; WHO 2001). However, some authors did not find a positive association between the consumption of seafood and/or shellfish and the urinary concentrations of As. In particular, Hsueh et al. (2002) found no differences in the urinary concentrations of the various As species before and after refraining from eating seafood for 3 days, respectively.

Moreover, despite the notorious toxic effects of arsenic in humans, it is still used in both agriculture and industry (Park et al. 2010; Kumaresan and Riyazuddin 2001). This large amount of inorganic arsenic-based pesticides has led to serious environmental arsenic contamination (Datta and Sarkar 2005).

Given the notorious adverse effects of arsenic exposure in humans, the US Environmental Protection Agency (EPA) banned the use of many inorganic arsenic-based pesticides during the late 1980s and early 1990s (Quazi et al. 2013).

Although some countries have issued documents to phase out organo-arsenical pesticides from the market, large agricultural sites contaminated by years of organo-arsenical pesticide application still exist. These agricultural lands might pose significant health risks in the present and in the future (Li et al. 2016). About that, contribution of transdermal route in overall As intake is hard to estimate and thus cannot be investigated in this study.

In our study, no association was found between the use of pesticides and urinary concentrations of As. There were significant differences between those who drank tap water and those who habitually drank bottled mineral water. The contamination of the main water supply remains a major source of exposure to inorganic As in many parts of the world (IARC 2004), despite the fact that in 1993, the WHO recommended that levels of As in drinking water should not exceed 10 μg/L (WHO 1993). On the other hand, a recent analysis of 40 different labels of bottled mineral water on sale in Italy demonstrated higher levels of total As than the legal limit in five of them (Signorile et al. 2007). In contrast, in the surveys of water in the Apulian aqueduct over the period 2004–2006, total As values were consistently below 1 μg/L.

Moriske et al. found higher concentrations of heavy metals in indoor air pollution in houses with coal burning and open fireplaces than those in homes with central heating (Moriske et al. 1996). In our study, we investigated the association between having a fireplace in the home and the urinary excretion of heavy metals, but did not find any association.

Overall, the biological monitoring data reveal high urinary concentrations of both heavy metals investigated, above all in Statte municipality.

However, in our study, it was not possible to correlate the biological monitoring data with the environmental data because the information collected by the official institutions and/or those in the literature were incomplete and only provided by the European Monitoring and Evaluation Programme (EMEP).

For the whole province of Taranto, the value of emissions of Pb into the atmosphere in 2009 was 38 tons, one of the highest in Europe, and the emissions of Hg in the same year was 510 kg. There are no available data concerning the emissions of the other metals. In the future, therefore, we believe it will be necessary to carry out an organized environmental monitoring program, taking into consideration all exposure routes to correlate the environmental concentrations of these metals with the biomonitoring results. However, our study suffers from some limitations due to the small population sample and data analysis. In fact, the questionnaire results could be influenced by subjects’ personal replies. Moreover, chromium analysis could be affected by redox reactions interference, during the ions determination (Jiang et al. 2013). Furthermore, for arsenic speciation, best sensitivity was shown for As(III) with respect to MMA, DMA, and As(V) (Moreno et al. 2000).

In any case, the data we obtained, which may be further confirmed by larger population studies, are sufficient to warrant the expectation that local and national institutions should be required to adopt preventive measures to reduce the environmental exposure of the general population to heavy metals, especially lead and chromium. Such actions could help to reduce the health risks, including those of a carcinogenic nature, posed to populations residing in areas with a known high environmental impact.

## Conclusions

The importance of investigating the exposure of the general population to As and Cr lies in their ubiquitous nature, since they are also widely distributed in nature, as well as in their harmful effects on human health. We conducted a study to evaluate the exposure to heavy metals in the industrial city of Taranto and the surrounding area in Southern Italy through biological monitoring techniques.

We measured the levels of chromium, inorganic arsenic, and its methylated metabolites in the urine samples of 279 subjects residing in Taranto and neighboring areas.

Our study results showed high urinary concentrations of both heavy metals investigated.

It would be appropriate to search the causes of this finding and deepen the impact of industrial plants present in that area. This is important in developing a comprehensive risk assessment and management program in order to adopt preventive measures to reduce environmental exposure of the general population to As and Cr, considering their toxicity and carcinogenicity.

Further epidemiological studies with larger samples and including environmental air quality data will be necessary to confirm our results.
